# Evoked potential changes in patients with Parkinson's disease

**DOI:** 10.1002/brb3.703

**Published:** 2017-04-07

**Authors:** Chunyan Liu, Yaping Zhang, Weiguo Tang, Binda Wang, Bona Wang, Songbin He

**Affiliations:** ^1^ Department of NeurologyZhoushan HospitalWenzhou Medical UniversityZhoushanChina

**Keywords:** brainstem auditory evoked potential, interpeak latency, latency, Parkinson's disease, visual evoked potential

## Abstract

**Objective:**

Patients with Parkinson's disease (PD) may have sensory dysfunction, and it can be more easily demonstrated through electrophysiologic testing. We aimed to explore whether the impairment of brainstem visual and auditory passageway exists in PD patients using visual evoked potential (VEP) and brainstem auditory evoked potential (BAEP) examinations.

**Methods:**

Forty‐two PD cases and thirty controls participated in the study. All subjects underwent the VEP and BAEP examinations. The N75, P100, N145 latencies and P100 amplitude of VEP, the latencies of waves I, III, V and the interpeak latencies (IPL) of waves I–III, III–V, I–V were measured.

**Results:**

The N75, P100, N145 latencies of VEP, but not the amplitude of P100, were significantly longer in patients with PD than the control group (*p *<* *.05). The latencies of wave III and wave V, the IPL of III–V and I–V were all significantly increased compared with control subjects while no significant difference was noted in waves I and I–III IPL.

**Conclusion:**

Our results found that brainstem visual and auditory passageway may be impaired in PD patients.

**Significance:**

VEP and BAEP can be served as sensitive measurements in helping prognosis and assessment the severity of the disease.

## 1 Introduction

PD is a common degenerative disease of central nervous system among middle‐aged populations. Its typical signs include distal resting tremor, bradykinesia, rigidity, and postural disturbances. Patients with PD may have sensory dysfunctions such as visual dysfunction, olfactory dysfunction, vestibular dysfunction, and pain. Most of these abnormalities are relatively subtle from a clinical point of view but can be more easily demonstrated through electrophysiologic or psychophysical testing.

The diagnosis of PD currently mainly depends on the identification of disease history, symptoms, and physical examination, which are difficult to be quantitative and objective. Because of the variability of PD symptoms, the severity and clinical staging of PD cannot be accurately assessed in clinical practice. The measurement of the evoked potential (EP) is a widely used noninvasive technique for studying the functional changes in neural conductive pathway of PD. Some studies demonstrated VEP or BAEP in patients with PD, but the results were inconsistent (Deng, Deng, Zhao, Yan, & Chen, 2006; Li, 2004; Venhovens, Meulstee, Bloem, & Verhagen, 2016; Vitale et al., 2012; Yylmaz et al., 2009). So our study explored whether the impairment of brainstem auditory and visual passageway exists in patients with PD using VEP and BAEP. It can imply the location of impairment was in the brainstem or the end organ and find its association with the clinical stage and severity of the disease.

## 2 Patients and methods

### 2.1 Subjects

This was a case–control study developed between October 2015 and July 2016. Forty‐two outpatients and inpatients including 18 males and 24 females were registered in the Department of Neurology, Zhoushan Hospital of Zhejiang Province, and enrolled as the PD group, written informed consent for research purposes. PD was diagnosed according to the United Kingdom Parkinson's Disease Society Brain Bank clinical diagnostic criteria. Thirty age‐matched healthy controls were included. Patients inclusion criteria were as follows: (1) without history of neurological disease or psychiatric disease; and (2) brain MRI showing normal image; patients exclusion criteria were as follows: (1) patients with dementia, severe anxiety, depression, psychosis, cerebrovascular illness, ophthalmologic, and auditory diseases; (2) secondary Parkinson's syndrome; (3) and Other systematic diseases such as asthma, chronic obstructive pulmonary disease, heart failure, and cardiac arrhythmia.

### 2.2 Methods

All enrolled patients underwent a series of detailed history examinations. Hamilton Anxiety and Depression Scale was used to evaluate the psychological state, and the mini‐mental state examination (MMSE) was used to test the intelligence. All the results of these scales were in normal range. Severity of disease was assessed by UPDRS‐III, and stage of PD was assessed by H&Y classification. All patients experienced these assessments and were between the H&Y stages 1 and 4 (mean 2.01). The ophthalmologic evaluations were performed at eye clinic and comprised a visual acuity test (Snellen table), an Ishihara colors test, a biomicroscopy, and an intraocular pressure measurement using the Goldmann applanation tonometer. The hearing tests included standard audiometric pure‐tone air and bone conduction testing. All patients, except two, were all on L‐dopa or a dopamine agonist therapy. Patients were assessed in the “on” state before the morning dose of the drug. The daily dose of dopa therapy was calculated by addition of a daily dose of levodopa and the dopaminergic agonists transcribed as “dose‐equivalent dopa” (Krack et al., 1998; Lozano et al., 1995). The mean body mass index (BMI) and years of education were also measured. The purpose of the detection should be explained to the patients in advance.

The VEP and BAEP were demonstrated by Evoked Potential Response Unit (Keypoint 4, Dantec™, Denmark) in a dim and quiet room in Zhoushan Hospital. VEP used a black‐white checkered‐board pattern on a television monitor with a dimension of 5×5 cm for every check. The patterns had a contrast of 60%, and the mean luminance was 300 cd/m^2^. Contrast is defined as the difference between the maximum and minimum luminance of adjacent vertical bands over their sum. The reversal rate was 3 Hz. The observer's distance was 100 cm from the screen. The recording electrode was in the midline and 5 cm above the inion with the reference 20 cm anterior to it. Viewing was monocular, and both eyes were tested separately in each subject. N75, P100, N145 latencies, and P100 amplitude were measured, and the averaged data of left and right eye in each subject were recorded. BAEP recording electrode was placed at Cz while the reference electrode at earlobe and the ground electrode on the forehead according to international standard 10–20 system. The short sound was used to stimulate the target ear with the interval of 0.1 ms, the frequency of which was 11.1 Hz, intensity was 105 db, superposition was 2048 times while the opposite one screened noisily, and then repeated for twice or more in the same way. Both ears were tested separately in each subject. The latencies of waves I, III, V and the interpeak latencies (IPL) of waves I–III, III–V, I–V were measured and the averaged data of contralateral and the ipsilateral ear were recorded.

Data analysis was performed using SPSS software (version 21.0, IBM, Chicago, IL, USA). Values were expressed as means ± standard deviation unless otherwise specified. Comparisons of baseline data between PD group and the control group were performed with the Student's *t* test. Chi‐square test was used to determine group distribution. The correlation of VEP and BAEP with age, years of education, UPDRS scores, disease duration, dopa dose was evaluated using Pearson's correlation test while the correlation of VEP and BAEP with H&Y classification was evaluated using Spearman rank correlation test. The correlation of dopa dose with age, UPDRS scores, disease duration was also demonstrated using Pearson's correlation test, while correlation of dopa dose with H&Y classification was evaluated using Spearman rank correlation test. *p* value < .05 was considered significant. Partial correlation analysis was used by controlling other covariates when correlation between two covariates was found.

## 3 Results

### 3.1 Quantitative analysis of participants

Forty‐two patients (84 eyes) with PD and 30 (60 eyes) age‐matched healthy controls were examined. Demographic and clinical assessment data for patient and control groups are presented in Table 1. The groups were not statistically different for age (*t* test: t = 0.25, *p *=* *.803), gender distribution (chi‐square test: χ^2^ = 0.002, *p *=* *.968), mean body mass index (*t* test: t = −0.05, *p *=* *.957), years of education (*t* test: t = 0.192, *p *=* *.867), and MMSE scores (*t* test: t = −0.141, *p *=* *.407).

**Table 1:** Demographic and clinical data of groups (Mean ± *SD*)

	PD	Control	*p*
*N*	42	30	
Age	69.24 ± 6.94	68.83 ± 6.54	0.803
Sex (male/female)	18/24	13/17	0.968
BMI	21.28 ± 2.51	21.32 ± 2.94	0.957
Education (years)	4.00 ± 3.57	3.83 ± 3.72	0.867
MMSE	24.45 ± 3.31	24.57 ± 3.50	0.407
Duration (years)	3.46 ± 2.47	–	
UPDRS	34.26 ± 17.51	–	
H&Y	2.01 ± 0.73	–	
Dopa dose	271.26 ± 163.24	–	

PD, Parkinson's disease; BMI, body mass index; UPDRS, Unified Parkinson Disease Rating Scale; H&Y, Hoehn and Yahr Scale; MMSE, mini‐mental state examination.

### 3.2 Abnormality of VEP

**Figure 1:**
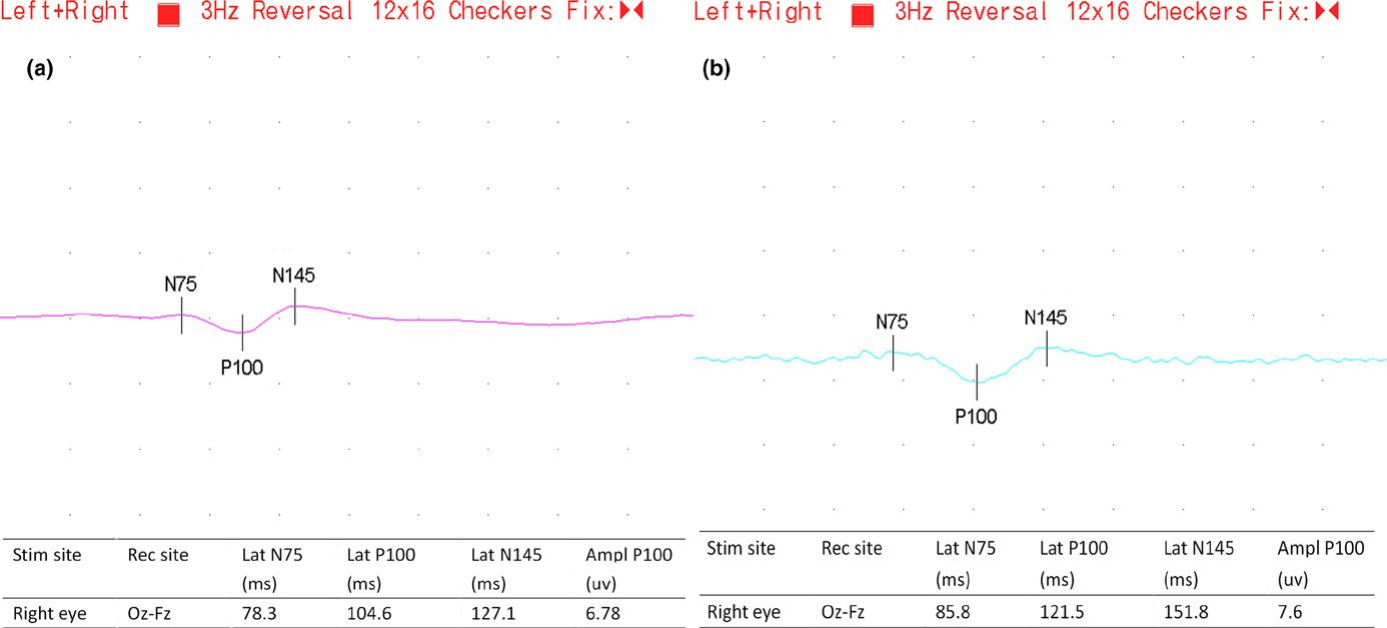
(a) VEP wave pattern of a healthy participant. (b) VEP wave pattern of a patient with PD

**Figure 2:**
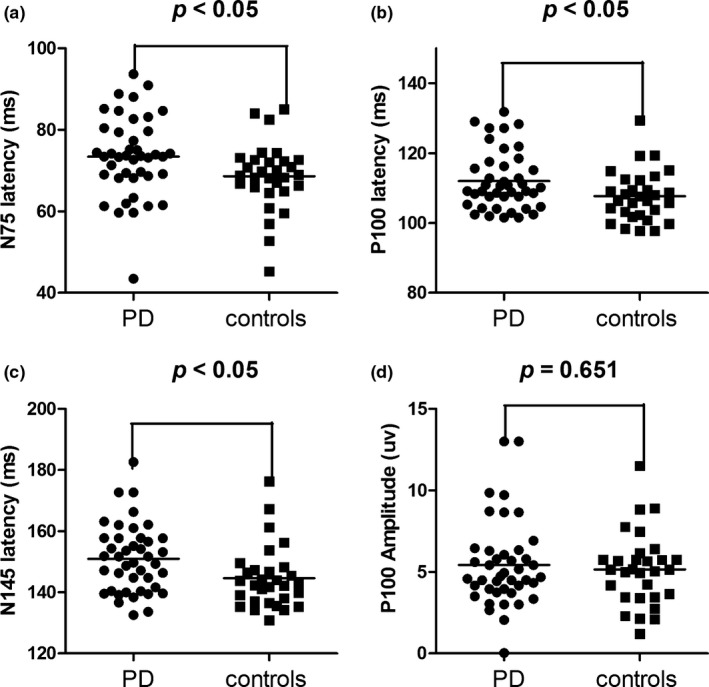
(a) Comparisons of the N75 latency between PD group and the controls. (b) Comparisons of the P100 latency between PD group and the controls. (c) Comparisons of the N145 latency between PD group and the controls. (d) Comparisons of the P100 amplitude between PD group and the controls

Figure 1 shows VEP wave patterns of a healthy individual in the control group and a patient with PD. Comparisons showed that the latency of N75, P100, N145, but not the amplitude of P100, was significantly longer in patients with PD than the control group (*p* all < .05) (Table 2) (Figure 2).

**Table 2:** Comparisons of VEP in patients with PD and healthy controls

Group	Latency (ms)			Amplitude (uv)
	N75	P100	N145	P100
PD	73.45 ± 9.86	112.01 ± 8.36	150.99 ± 11.12	5.43 ± 2.63
Control	68.61 ± 8.28	107.71 ± 7.22	144.60 ± 10.14	5.16 ± 2.24
*p*	0.032^a^	0.026^a^	0.015^a^	0.651

a *p* < .05.

*p* < .01.

### 3.3 Abnormality of BAEP

**Figure 3:**
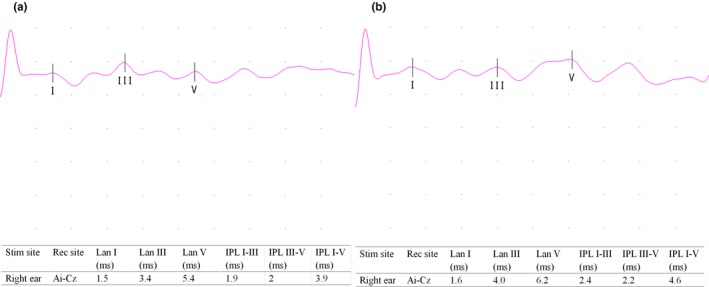
(a) BAEP wave pattern of a healthy participant. (b) BAEP wave pattern of a patient with PD

**Figure 4:**
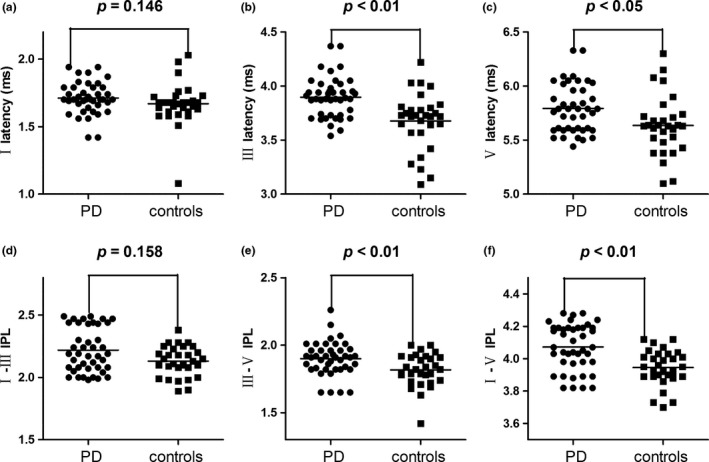
(a) Comparisons of the wave I latency between PD group and the controls. (b) Comparisons of the wave III latency between PD group and the controls. (c) Comparisons of the wave V latency between PD group and the controls. (d) Comparisons of the I–III IPL between PD group and the controls. (e) Comparisons of the III–V IPL between PD group and the controls. (f) Comparisons of the I–V IPL between PD group and the controls

Figure 3 shows BAEP wave patterns of a healthy individual in the control group and a patient with PD. As seen in Table 3, patients with PD showed significantly increased latencies in waves III and V compared with control subjects (*p *=* *.001, *p *=* *.010, respectively) (Table 3) but there was no significant difference in latencies of wave I. In addition, there were significant increases in III–V, I–V IPLs for PD patients compared with control subjects (*p *=* *.005, *p *<* *.001, respectively) (Table 3) (Figure 4) although no significant difference noted in I–III IPL.

**Table 3:** Comparisons of latency (ms) of BAEP in patients with PD and healthy controls

Group	Latency of the dominant wave			Interwave interval of the dominant wave		
	I	III	V	I–III	III–V	I–V
PD	1.71 ± 0.10	3.85 ± 0.19	5.79 ± 0.21	2.18 ± 0.16	1.92 ± 0.15	4.08 ± 0.14
Control	1.67 ± 0.16	3.68 ± 0.26	5.64 ± 0.27	2.13 ± 0.12	1.82 ± 0.12	3.95 ± 0.11
*p*	0.146	0.001^b^	0.010^a^	0.158	0.004^b^	<0.001^b^

a *p* < .05.

b *p* < .01.

### 3.4 The correlation of VEP and BAEP with others

**Figure 5:**
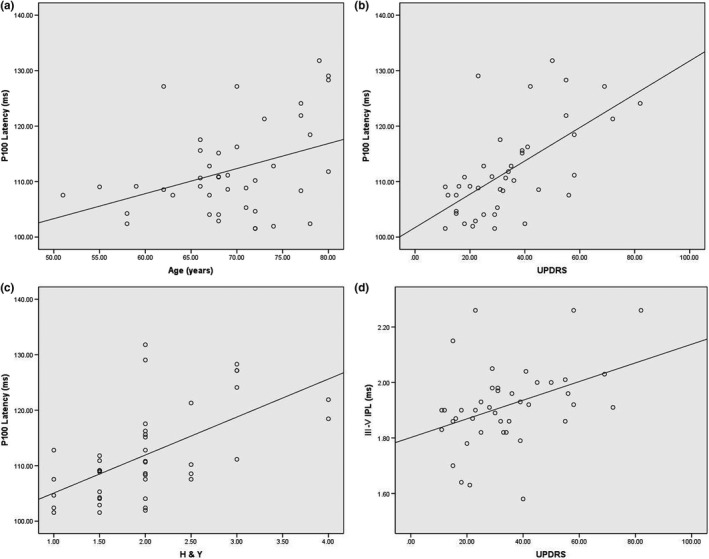
(a) Correlation of the P100 latency and age of PD. (b) Correlation of the P100 latency and UPDRS scores of PD. (c) Correlation of the P100 latency and Hoehn and Yahr of PD. (d) Correlation of the III–V IPL and UPDRS scores of PD

The P100 latency of VEP was positively correlated with the ages (r = .375, *p *=* *.014) (Table 4, Figure 5a), UPDRS score (r = .629, *p *<* *.001) (Table 4, Figure 5b), and H&Y classification (r = .597, *p *<* *.001) (Table 4, Figure 5c) while it did not show any relation between P100 latency with duration of disease and dopa dose (Table 4) in patients with PD. Partial correlation analysis showed that when controlling the UPDRS and H&Y covariates, there is no correlation between P100 latency and ages. However, when controlling the age covariate, the results changed little. The III‐V IPL of BAEP was only found positively correlated with UPDRS score (r = .398, *p *=* *.009) (Table 4, Figure 5d) in patients with PD. The dopa dose was positively correlated with UPDRS (r = .370, *p *=* *.019) and duration (r = .644, *p *<* *.001) of PD. Partial correlation analysis showed that when controlling the duration covariate, there is no correlation between dopa dose and UPDRS. On the contrary, when controlling the UPDRS covariate, the relation between dopa dose and duration did not changed.

**Table 4:** Correlations between VEP, BAEP, UPDRS scores, H&Y stages, duration of disease, and dopa dose in PD

	Age	UPDRS	H&Y	Duration	Dopa dose
P100 latency					
Correlation coefficient (r)	0.375	0.629	0.582	0.210	0.193
*p*	0.014^a^	<0.001^b^	<0.001^b^	1.181	0.232
III‐V intervals					
Correlation coefficient (r)	0.108	0.398	0.176	0.193	0.161
*p*	0.494	0.009^b^	0.264	0.220	0.321
Dopa dose					
Correlation coefficient (r)	0.206	0.370	0.245	0.644	–
*p*	0.202	0.019^a^	0.127	<0.001^b^	–

PD, Parkinson's disease; UPDRS, Unified Parkinson's Disease Rating Scale; H&Y, Hoehn and Yahr Scale.

a *p* < .05.

b *p* < .01.

## 4 Discussion

Most of the sensory abnormalities linked to PD are demonstrable in the very early clinical phase of the illness and are presumably present in the preclinical phase of PD as well. The neuro‐electrophysiologic measurements such as VEP and BAEP may provide a widely used non‐invasive technique to evaluate the functional changes of sensory conductive pathway of patients with PD.

It is worth noted that VEP measures the integrity of the entire visual pathway. The changes in these potentials in PD may reflect the widespread nature of the biochemical disorder affecting both retina and central nervous system. P100 latency is less likely to be affected by dopaminergic drugs and seems to be a more sensitive measure. As to BAEP, wave I is associated with the electric activity of outer cranium segment. Wave III is associated with the medial super‐olive nucleus or cochlea nucleus. Wave V is associated with lateral lemniscus nucleus colony and may be the electric activities of central nucleus in hypothalamus. I–III IPL represents the conduction time from auditory nerve to inferior pons while III–V IPL represents the conduction time from inferior pons to inferior midbrain.

From the result of present study, the N75, P100, N145 components of VEP had more prolonged latency in patients with PD than controls, which was inconsistent with some other studies (Li, 2004; Miri, Glazman, Mylin, & Bodis‐Wollner, 2016). But the amplitude of P100 was not significantly different between the two groups. From the result of Pearson's correlation test and partial correlation analysis, we may draw a conclusion that the P100 latency was positively correlated with UPDRS score and H&Y classification. The main BAEP abnormalities occurred in III and V latency in most patients with PD, which were significantly different from those in the normal control group. III–V and I–V IPL were obviously different between the two groups, whereas there was no obvious difference in I–III IPL. What's more, the III–V IPL of BAEP was only found positively correlated with UPDRS score. Similar to our study, Tachibana et al. have reported statistically significant increase in V wave peak latency and I–V, III–V IPLs for patients with PD (Tachibana, Takeda, & Sugita, 1989). On the other hand, Tsuji et al. (Tsuji, Muraoka, Kuroiwa, Chen, & Gajdusek, 1981), Prasher and Bannister (Prasher & Bannister, 1986) have reported normal BAEPs in patients with PD.

The exact etiology and pathogenesis of PD remain elusive while it is widely accepted that PD is caused by progressive loss of dopaminergic neurons in the substantia nigra. In our study, VEP and BAEP reflected functional impairment in the brainstem visual and auditory system in patients with PD. The abnormal VEP is incapable of differentiating impaired macular function from impaired superior brainstem function. We could not exclude the retinal impairment as someone had found structural damage in the retina of patient with PD using optical coherence tomography (OCT) (Inzelberg, Ramirez, Nisipeanu, & Ophir, 2004). Abnormal BAEP suggested the existence of impairment of superior brainstem in the auditory conduction pathway of patients with PD. According to what we found above, the impairment of upper brainstem may exist in both visual and auditory conductive pathway. It also indicated that the impaired VEP was affected by clinical stage and severity of PD while impaired BAEP was affected by severity of PD. Therefore, the VEP and BAEP may be an effective method of assessment the severity of PD.

Some studies found that substitution of dopaminergic drugs was shown to reverse VEP delays in patients with PD(Barbato, Rinalduzzi, Laurenti, Ruggieri, & Accornero, 1994; Bodis‐Wollner & Yahr, 1978; Onofrj, Ghilardi, Basciani, & Gambi, 1986) while someone observed no improvement after levodopa (Nightingale, Mitchell, & Howe, 1986). Podoshin et al. Podoshin, Ben‐David, Fradis, and Pratt (1987) and Fradis et al. Fradis et al., (1988) found no significant differences of BAEP between patients with PD under dopaminergic drugs treatments and those without treatment. Our study found no significant correlation between EP and dopa dose. It may be explained that the dopa dose had positive correlation with clinical duration as we had found. As PD developing, more dopa was used. So we were not able to reveal the real correlation between the EP and dopaminergic drugs as the duration and severity of PD were different.

## 5 **Conclusion**

Above all, our results found that brainstem visual and auditory passageway may be impaired besides the involvements of substantia nigra and striatum in patients with PD. Both extra corticospinal tract and the sensory system are involved in patients with PD. VEP and BAEP can be effective tools for detecting the functional changes of brainstem in patients with PD and may help in prognosis and assessment the severity of the disease. Further research is needed to explore the mechanisms underlying this relationship.

## Conflicts of Interest

None.
